# Post-Stroke Psychosis Following Basilar Artery Occlusion: A Case Report

**DOI:** 10.1192/j.eurpsy.2025.1234

**Published:** 2025-08-26

**Authors:** H. J. Gomes, M. Pires, J. R. Gomes

**Affiliations:** 1 Unidade Local de Saúde do Nordeste, Bragança; 2 Unidade Local de Saúde da Guarda, Guarda, Portugal

## Abstract

**Introduction:**

Approximately 16 million individuals experience a first-time stroke annually, with vertebrobasilar (VB) strokes accounting for 20% of all strokes and transient ischemic attacks. Neuropsychiatric symptoms affect at least 30% of stroke survivors and are associated with low quality of life, an increase in the burden of caregiving, and impaired functional status. These complications can range from mood disorders to psychosis. Psychosis is a relatively rare complication after stroke but is among the most serious of the poststroke syndromes. However, there are no specific guidelines for its treatment.

**Objectives:**

To describe a case of psychosis following a vertebrobasilar stroke and review the relevant literature on the topic.

**Methods:**

We report a case of post-stroke psychosis in a man with no prior psychiatric history and conduct a non-systematic review of the literature.

**Results:**

A 46-year-old man with a history of dyslipidemia and testicular cancer 13 years prior and no previous psychiatric history was admitted to the Stroke Unit under the care of Internal Medicine and Neurology due to a vertebrobasilar stroke. Liaison Psychiatry was called to assess the patient on the 27th day of hospitalization due to visual hallucinations. On examination, the patient presented with a suspicious facial expression, persecutory and infidelity delusions, and both visual and auditory hallucinations. No other psychopathological changes were observed. The case was discussed with the Neurology team, and the patient underwent laboratory tests, a new head computed tomography, and an electroencephalogram, none of which showed recent significant abnormalities. The patient was started on olanzapine 5 mg daily. The patient was reassessed after one week and continued to have the described psychotic symptoms, leading to an increase in the olanzapine dose to 10 mg. After two weeks of treatment, there was a noticeable reduction in the psychotic symptoms.

**Conclusions:**

Psychosis is a possible complication of stroke and is associated with impairment and increased mortality. According to the literature, strokes in the vertebrobasilar territory can cause hallucinations. Guidelines for managing poststroke psychosis are currently lacking, and to assure evidence-based care, further research is needed. This case underscores the importance of early detection and intervention, emphasizing the need for a multidisciplinary approach to managing post-stroke psychosis.

**Disclosure of Interest:**

None Declared

